# Identification and analysis of cellular senescence-associated signatures in diabetic kidney disease by integrated bioinformatics analysis and machine learning

**DOI:** 10.3389/fendo.2023.1193228

**Published:** 2023-06-16

**Authors:** Yuanyuan Luo, Lingxiao Zhang, Tongfeng Zhao

**Affiliations:** ^1^ Department of Endocrinology, The Sixth Affiliated Hospital, Sun Yat-sen University, Guangzhou, China; ^2^ Department of Endocrinology, The Third Affiliated Hospital of Southern Medical University, Guangzhou, China

**Keywords:** diabetic kidney disease, cellular senescence, mitochondrial function, immune cell infiltration, bioinformatics analysis, machine learning

## Abstract

**Background:**

Diabetic kidney disease (DKD) is a common complication of diabetes that is clinically characterized by progressive albuminuria due to glomerular destruction. The etiology of DKD is multifactorial, and numerous studies have demonstrated that cellular senescence plays a significant role in its pathogenesis, but the specific mechanism requires further investigation.

**Methods:**

This study utilized 5 datasets comprising 144 renal samples from the Gene Expression Omnibus (GEO) database. We obtained cellular senescence-related pathways from the Molecular Signatures Database and evaluated the activity of senescence pathways in DKD patients using the Gene Set Enrichment Analysis (GSEA) algorithm. Furthermore, we identified module genes related to cellular senescence pathways through Weighted Gene Co-Expression Network Analysis (WGCNA) algorithm and used machine learning algorithms to screen for hub genes related to senescence. Subsequently, we constructed a cellular senescence-related signature (SRS) risk score based on hub genes using the Least Absolute Shrinkage and Selection Operator (LASSO), and verified mRNA levels of hub genes by RT-PCR in vivo. Finally, we validated the relationship between the SRS risk score and kidney function, as well as their association with mitochondrial function and immune infiltration.

**Results:**

The activity of cellular senescence-related pathways was found to be elevated among DKD patients. Based on 5 hub genes (LIMA1, ZFP36, FOS, IGFBP6, CKB), a cellular senescence-related signature (SRS) was constructed and validated as a risk factor for renal function decline in DKD patients. Notably, patients with high SRS risk scores exhibited extensive inhibition of mitochondrial pathways and upregulation of immune cell infiltration.

**Conclusion:**

Collectively, our findings demonstrated that cellular senescence is involved in the process of DKD, providing a novel strategy for treating DKD.

## 1 Introduction

Diabetic kidney disease (DKD) is one of the most common complications of diabetes and remains the leading cause of end-stage renal disease (ESRD) in the world population (1). The current treatments for DKD are largely confined to optimal glucose and blood pressure control, as well as renin–angiotensin–aldosterone system blockades, which have limited therapeutic efficacy (1). It is therefore imperative to gain a deeper understanding of the pathogenesis of DKD, identify reliable biomarkers for high-risk patients, and develop novel therapeutic approaches to prevent or reverse the progression of DKD.

Cellular senescence is a cellular state that involves a halt in proliferation, resulting in inflammation, impaired tissue repair, irreversible tissue damage, and organ dysfunction (2). Recent research has shown that renal parenchymal cells are induced to undergo cellular senescence in the context of diabetes, by various pathogenic stimuli, including hyperglycemia (3) and the accumulation of advanced glycation end products (AGEs) (4). This leads to the deterioration of kidney function mainly by mediating a complex pro-inflammatory response called senescence-associated secretory phenotype (SASP) (3). The SASP includes the secretion of various molecules including cytokines, chemokines, and growth factors, and has been suggested as a possible source of inflammatory factors in DKD (5). However, the linkage of cellular senescence and DKD is complex and not yet fully understood at present. Therefore, it is vital to clarify the role and mechanism of cellular senescence in the pathogenesis of DKD.

To systematically assess the correlations between cellular senescence and the pathogenesis of DKD, we developed a cellular senescence-related signature (SRS) risk score using biomarkers that are closely associated with cellular senescence, and evaluated its potential significance in predicting renal function. Subsequently, we grouped patients according to the SRS risk score and compared mitochondrial function and immune cell infiltration between the high and low the SRS score groups. Our study sheds new light on the regulatory mechanisms of cellular senescence in the process of DKD.

## 2 Materials and methods

### 2.1 Data acquisition

The microarray data of the mRNA expression profile related to DKD are retrieved from the Gene Expression Omnibus (GEO) database (http://www.ncbi.nlm.nih.gov/geo/). More details of the collected datasets are presented in **Supplementary Table 1**. Cellular senescence-related pathways were derived from the Molecular Signatures Database (6). A total of 279 cellular senescence-associated genes were downloaded from the CellAge database https://genomics.senescence.info/cells/) (7). Clinical data for DKD patients were downloaded from the Nephroseq v5 online database (http://v5.nephroseq.org) (8). The mitochondrial genes and pathways were obtained from the MitoCarta3.0 database (http://www.broadinstitute.org/mitocarta) (9) and the Reactome database (https://reactome.org) (10). Our workflow is illustrated in **Supplementary Figure 1.**

### 2.2 Data preprocessing

To combine the data from the above 5 datasets, we first removed batch effects using the surrogate variable analysis (SVA) algorithm (11). The distribution patterns of samples were visualized by box plots and principal component analysis (PCA).

### 2.3 Pathway and functional enrichment analysis

Kyoto Encyclopedia of Genes and Genomes (KEGG) (12) and Gene Ontology (GO) (13) enrichment analyses were applied using the R package clusterProfiler (14). Gene set enrichment analysis (GSEA) (15) was also performed to identify the underlying pathways; the threshold for significant terms was adjusted *p*-value < 0.05.

### 2.4 Gene set variation analysis

Gene set variation analysis (GSVA) is a nonparametric unsupervised analysis method mainly used to evaluate the gene set enrichment results of sequencing; GSVA allows the assessment of potential changes in pathway activity in each sample. The GSVA package in R software was used for the analysis, and the enrichment scores of pathways in all samples were calculated (16).

### 2.5 Construction of the co-expression network and key module identification by weighted gene co-expression network analysis

We used the Weighted Gene Co-Expression Network Analysis (WGCNA) algorithm (17) to screen for cellular senescence-associated module genes based on the enrichment scores of pathways obtained from GSVA. The “goodSamplesGenes” function was utilized to identify and remove outliers. To ensure that the co-expression network followed a scale-free distribution, we calculated a soft-thresholding power with the “pickSoftThreshold” function. We used the dynamic tree-cutting method to identify different modules, setting a minimum number of 100 genes per module. The module with the highest correlation with cellular senescence-associated pathways was identified as the senescence-associated module genes.

### 2.6 Identification of intersection genes

The differentially expressed genes (DEGs) between the normal and DKD groups were implemented via the “limma” R package (18) and visualized with heatmaps and volcano plots by the “ggplot2” package in R software (19). The intersection genes were obtained by the overlapping DEGs between control and DKD, cellular senescence-associated genes from the CellAge database, and module genes using a Venn diagram.

### 2.7 Screening hub genes of DKD by machine learning

Two machine learning algorithms, Support Vector Machine-Recursive Feature Elimination (SVM-RFE) (20) and Random Forest (RF) algorithms (21), were employed to further screen the cellular senescence-associated signature genes in intersection genes. SVM-RFE is a sequence backward selection algorithm, which has superior classification performance for high-dimensional datasets (22). The SVM-RFE algorithm was implemented using the “e1071”, “kernlab”, and “caret” packages in R software for feature dimensionality reduction. The RF algorithm is an ensemble method that combines many decision trees and makes a single decision on behalf of the ensemble by combining the results of multiple classifiers together. The RF algorithm was implemented using the “randomForest” package in R software. Ultimately, genes overlapping among the machine learning algorithms were regarded as hub genes. We also developed a receiver operating characteristic (ROC) curve to assess the predictive capacity of the hub genes. The area under the ROC curve (AUC) value was calculated using the pROC package (23) to estimate the predictive utility of the hub genes.

### 2.8 Development and validation of the SRS based on the LASSO

The Least Absolute Shrinkage and Selection Operator (LASSO) (24) was used to construct optimal SRS in DKD. The risk score formula was as follows: $\sum_{\text{i}=1}^{n}\beta i*\;\exp(\text{i})$ , where exp represented the gene expression value, and β represented the LASSO coefficient.

### 2.9 Analysis of immune cell proportion

GSVA based on the single-sample gene set enrichment analysis (ssGSEA) algorithm was used to quantify the infiltration of 28 immune cell types (15).

### 2.10 Animal experimental design

All mouse studies were conducted according to protocols approved by the Ethics Committee of The Sixth Affiliated Hospital, Sun Yat-sen University Male C57BL/6 mice aged 4 weeks old were used for experiments. High-fat diet and streptozotocin (HFD/STZ) were used to produce the DKD model following the previous study (25). The mice were randomly divided into two groups: the control group and the HFD/STZ group. The control group was fed a normal chow diet, and the HFD/STZ group was given HFD. After 4 weeks, the mice in the HFD/STZ group were intraperitoneally injected with a dose of STZ (100 mg/kg) to induce diabetes. One week later, the mice were considered diabetic if their random blood glucose levels exceeded 16.7 mmol/L. The control group received citrate buffer and was processed in parallel with the diabetic mice. Mice were sacrificed 16 weeks later.

### 2.11 Histology

Paraffin-embedded kidney sections were stained using commercial kits (Servicebio, Wuhan, China) for hematoxylin and eosin (H&E) staining, periodic acid–Schiff (PAS) staining, and Masson’s trichrome staining.

### 2.12 RT-PCR

At the termination of the animal experiment, we extracted total RNA from the kidney tissues of the mice using RNAiso Plus (Takara, Otsu, Japan). Then, reverse transcription was performed to synthesize cDNA (Novoprotein, Shanghai, China). The polymerase chain reaction was conducted using NovoStart SYBR qPCR SuperMix Plus (Novoprotein, Shanghai, China). The primer sequences are shown in **Supplementary Table 2**.

### 2.13 Correlation analysis between two matrices

Correlations between the 5 hub genes, the SRS risk score, and mitochondrial-related gene set were calculated using the Mantel test. The “cowplot” R package was used to create a graphical display of any correlations and their combinations.

### 2.14 Statistical analysis

All statistical tests were implemented utilizing R version 4.2.1 (https://www.r-project.org/) and GraphPad Prism 8.0. Wilcoxon or Student’s *t*-test was utilized for analyzing the difference between the two groups. The correlation between the variables was determined using Pearson’s or Spearman’s correlation test. All statistical *p* values were two-sided, and *p* < 0.05 was regarded as statistical significance.

## 3 Results

### 3.1 Removal of batch effects

After eliminating the batch effects by the SVA algorithm (11), we merged and normalized 5 DKD datasets. Box plots (**Supplementary Figures 2A, C**) and principal component analysis (PCA) (**Supplementary Figures 2B, D**) were used to visualize the distribution patterns between normal and DKD samples. The results confirmed that the batch effects had been successfully removed.

### 3.2 Identification of DEGs and enrichment analysis between control and DKD

**Figure 1:**
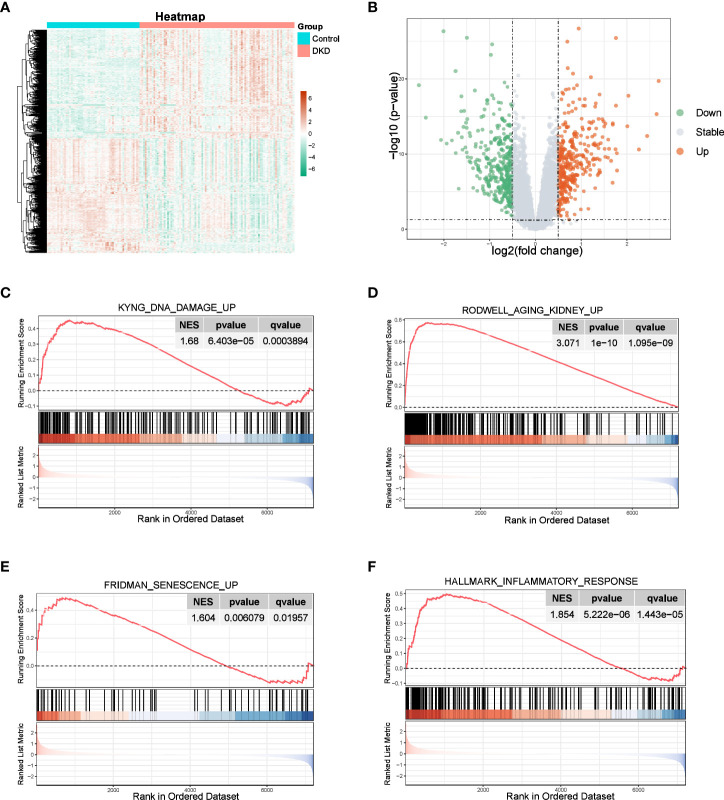
Identification of DEGs and enrichment analysis. **(A, B)** Heatmap and the volcano plot of the DEGs between normal and DKD groups. **(C–F)** GSEA of senescence-associated pathways.

We observed a significant distinction between normal and DKD samples, with 687 DEGs identified, including 334 upregulated genes and 352 downregulated genes, as shown in the volcano plot and heatmap (**Figures 1A, B**). GSEA results revealed that the “KYNG_DNA_DAMAGE_UP”, “AGING_KIDNEY_UP”, and “FRIDMAN_SENESCENCE_UP” pathways were activated (**Figures 1C–E**), all of which are associated with cellular senescence. Furthermore, since the senescence process is typically accompanied by an inflammatory response (5), we also observed activation of the “HALLMARK_INFLAMMATORY_RESPONSE” pathway within the hallmark gene sets of the DKD group (**Figure 1F**). These findings indicate that the senescence process plays a role in the development of DKD.

### 3.3 Analysis of module closely related to cellular senescence in DKD

**Figure 2:**
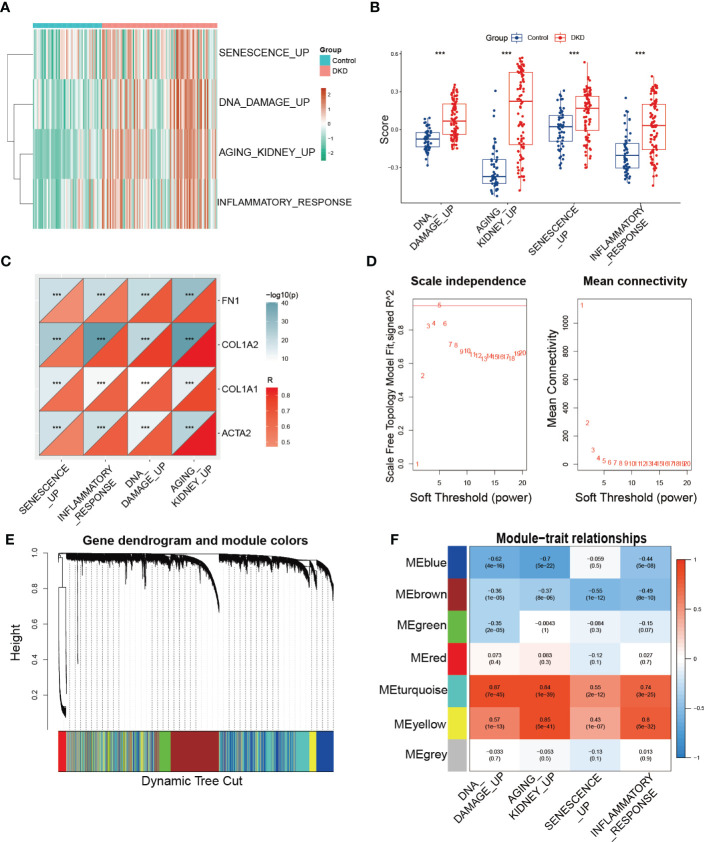
Identification of senescence-associated module genes in DKD. **(A, B)** GSVA of senescence-associated pathways demonstrated by heatmap and box plots in the control and DKD groups. **(C)** The correlation analysis between the score of senescence-associated pathways based on GSVA and fibrotic markers (*FN1, COL1A2, COL1A1*, and *ACTA2*). **(D)** Scale-free exponent and average connectivity for each soft threshold. **(E)** Dendrogram of gene clusters, with different colors representing different modules. **(F)** Heatmap of the correlations between module eigengenes and senescence-associated pathways. *** *p* < 0.001.

Furthermore, we utilized GSVA to calculate the enrichment score of the aforementioned senescence-associated pathways to evaluate potential changes in pathway activity across each sample, revealing that senescence-associated pathway activity was elevated in the DKD group (**Figures 2A, B**). We then performed correlation analysis to validate the association between senescence-associated pathway activity and renal fibrotic markers, demonstrating a strong positive correlation between pathway activity and the expression of fibrotic genes, such as *FN1, COL1A2, COL1A1*, and *ACTA2* (**Figure 2C**). Subsequently, we constructed a coexpression network using the enrichment score of senescence-associated pathways obtained via GSVA. We first calculated a soft threshold and established a scale-free topology model, setting the soft threshold to 5 (**Figure 2D**). The genes in the expression profile were then clustered (**Figure 2E**), with the yellow and turquoise modules displaying the strongest correlation with senescence-associated pathways (**Figure 2F**). Ultimately, we identified 2,938 module genes through WGCNA, which were identified as senescence-associated module genes in DKD.

### 3.4 Hub genes identified by machine learning models

**Figure 3:**
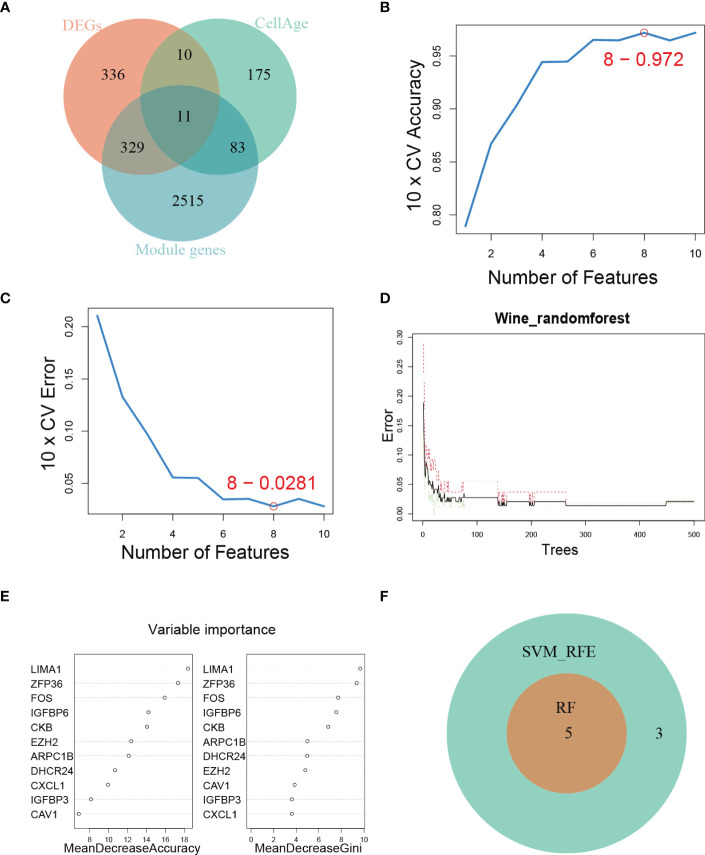
Hub genes identification. **(A)** Venn diagram showing the intersection of genes shared by WGCNA, DEGs, and genes from the CellAge database. **(B, C)** SVM-REF algorithm for feature selection. **(D)** RF algorithm demonstrating the relationship between the number of trees and error rate. **(E)** Ranking of genes based on their relative importance using the RF algorithm. **(F)** Venn diagram showing the hub genes shared by SVM-RFE and RF algorithms.

To comprehensively characterize the expression pattern of cellular senescence-related genes, we utilized the CellAge database to obtain 279 human genes related to cellular senescence. We then intersected these genes with senescence-associated module genes and DEGs between control and DKD using a Venn diagram, which revealed 11 overlapping genes (**Figure 3A**). To further narrow down the range of key senescence-related genes, we subjected these 11 genes to machine learning analysis. SVM-RFE and RF models were independently established based on the control and DKD groups. The SVM-RFE algorithm identified 8 feature genes (**Figures 3B, C**), while the RF algorithm generated a sequence of the 11 genes (**Figures 3D, E**). Finally, we identified the 5 most important explanatory variables (*LIMA1, ZFP36, FOS, IGFBP6*, and *CKB*) from the machine learning models as hub genes (**Figure 3F**).

### 3.5 Construction of SRS risk score based on the 5 hub genes

**Figure 4:**
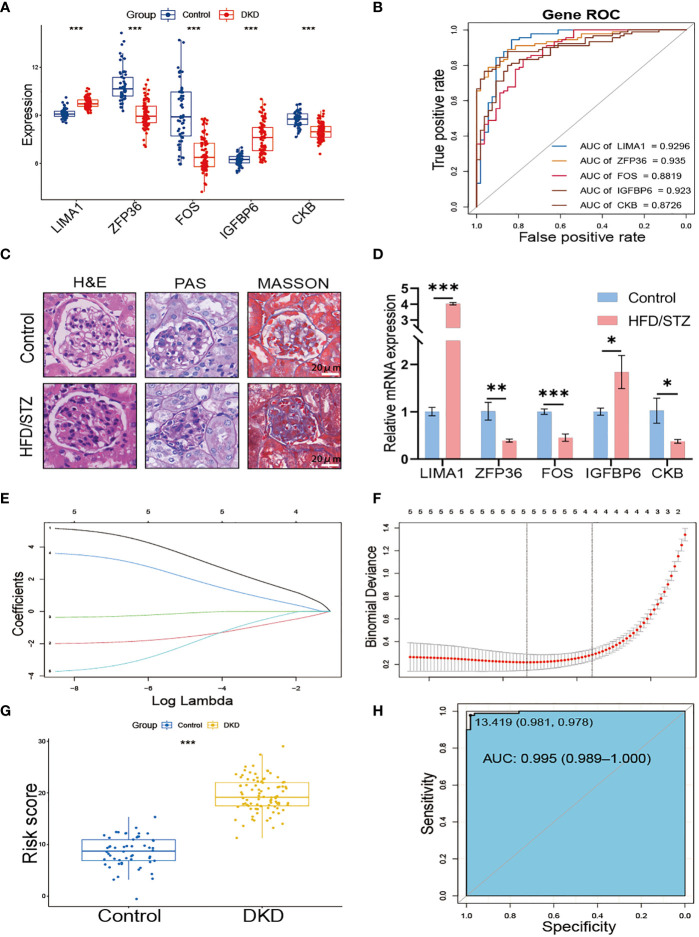
Construction of SRS risk score based on hub genes. **(A)** The expression of 5 hub genes displayed in box plots from control and DKD patients. **(B)** ROC curve of the 5 hub genes in DKD. **(C)** Representative images of H&E, PAS, and MASSON staining from control and HFD/STZ mice. **(D)** RT-PCR analysis of kidney *LIMA1, ZFP36, FOS, IGFBP6*, and *CKB* expression from control and HFD/STZ mice (*n* = 3). **(E)** Distribution of LASSO coefficients for differential genes. **(F)** Ten-time cross-verification for tuning parameter selection in the LASSO model. **(G)** The box plots of the SRS risk score between control and DKD patients. **(H)** ROC curve of the SRS risk score in DKD. * *p* < 0.05, ** *p* < 0.01, *** *p* < 0.001.

We further verified the expression of the 5 hub genes in the kidney of the normal control and DKD groups (**Figure 4A**). Additionally, we performed the ROC curve to evaluate the diagnostic efficacy of the 5 hub genes in DKD; the ROC curve indicated that all of the hub genes were highly accurate diagnostic markers for DKD (AUC between 0.8726 and 0.935) (**Figure 4B**). Subsequently, we utilized HFD/STZ to construct a DKD mouse model to verify the expression of the hub genes. Firstly, we validated the successful construction of the DKD model through histological analysis, which revealed that compared to the control group, the HFD/STZ group exhibited greater mesangial expansion and irregular thickening of the glomerular membrane. Additionally, Masson staining showed the formations of blue-stained extracellular collagen in the glomerulus (**Figure 4C**). Next, we performed RT-PCR to validate the expression of the hub genes in mouse kidney tissues. Our results indicated that the mRNA abundance of *LIMA1* and *IGFBP6* was higher in the HFD/STZ group, while that of *ZFP36*, *FOS*, and *CKB* was lower in the HFD/STZ group (**Figure 4D**), consistent with our bioinformatics analysis. Using the 5 hub genes, we employed LASSO to determine the number of factors by introducing shrinkage penalties and limiting the coefficients. Through continuous selection and simulation of the number of features, we established an SRS risk score for DKD patients (**Figures 4E, F**). The explicit formula of the SRS risk score was as follows: *LIMA1* expression * (3.792075646) + *ZFP36* expression * (−1.673662939) + *FOS* expression * (−0.150739822) + *IGFBP6* expression * (2.350866436) + *CKB* expression * (−2.398663582). We have further validated the diagnostic efficiency of the SRS risk score in DKD patients and found that it is highly effective for diagnosis (AUC = 0.995) (**Figures 4G, H**).

### 3.6 Validation of the role of the SRS risk score in DKD

**Figure 5:**
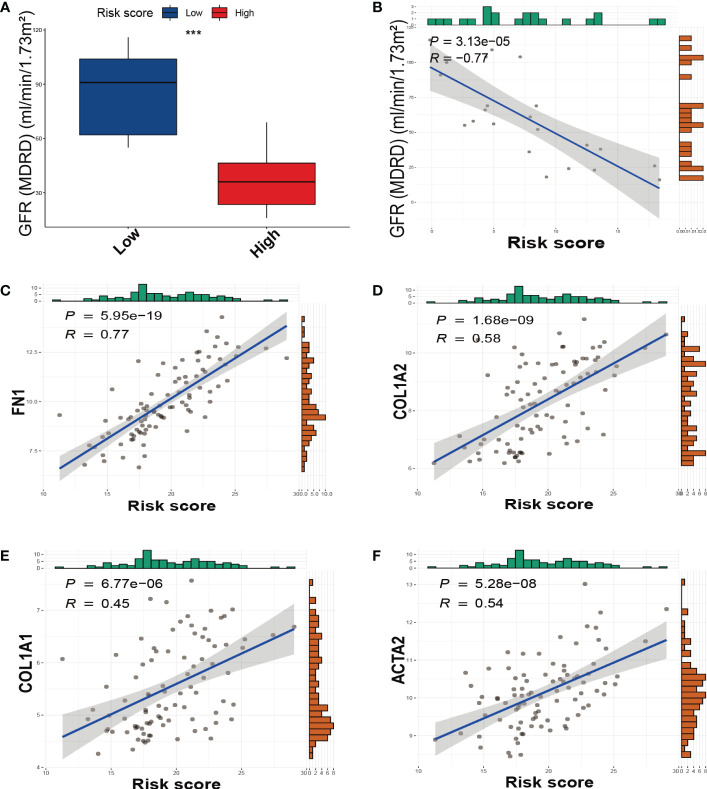
The relationship between the SRS risk score and GFR, fibrotic markers. **(A)** The box plots of GFR between the low-risk and high-risk groups. **(B)** The correlation analysis between the SRS risk score and GFR. **(C–F)** The correlation analysis between the SRS risk score and the expression of fibrotic markers (*FN1, COL1A2, COL1A1*, and *ACTA2*). *** *p* < 0.001.

We divided DKD patients into low-risk and high-risk groups based on the median of the SRS risk score. Subsequently, we examined the association between the risk score and renal function using clinical data from the Nephroseq v5 online tool. As depicted in **Figure 5A**, the high-risk group demonstrated a significantly lower glomerular filtration rate (GFR) than the low-risk group, and there was a negative correlation between the risk score and GFR levels (**Figure 5B**). We also investigated the link between the risk score and the expression of fibrotic genes. As illustrated in **Figures 5C–F**, the risk score exhibited a positive correlation with the expression of *FN1, COL1A2, COL1A1*, and *ACTA2.* These findings collectively validate the effectiveness of the SRS risk score as a means of assessing renal function and the degree of renal fibrosis.

### 3.7 Identification of DEGs grouped by the SRS risk score

**Figure 6:**
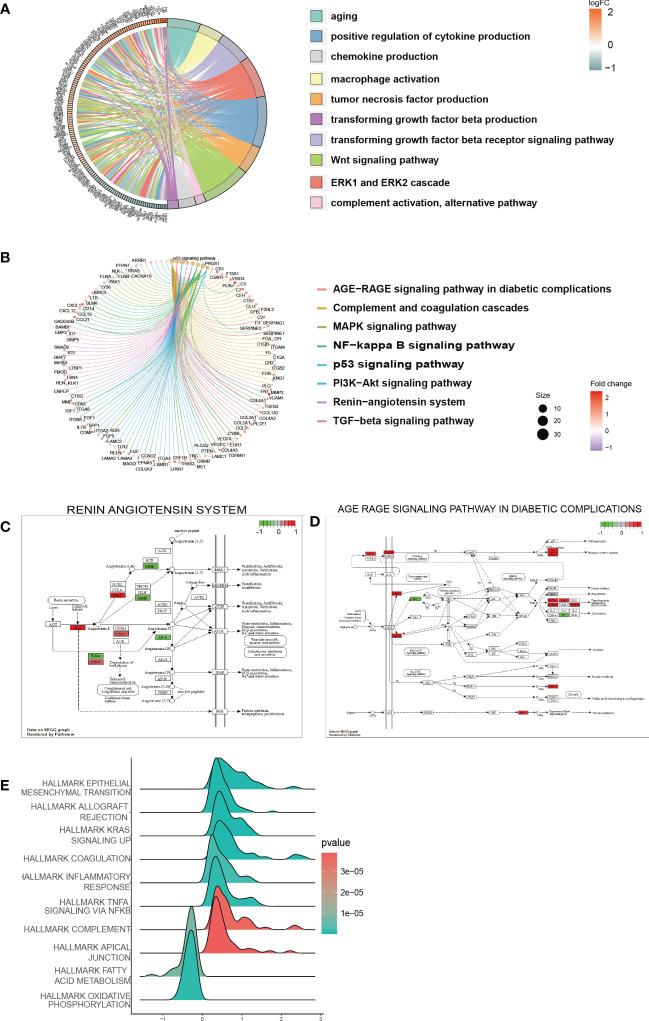
Function and pathway enrichment analysis of DEGs in the low-risk and high-risk groups. **(A)** GO terms enriched in the DEGs between the low-risk and high-risk groups. **(B)** KEGG pathway between the low-risk and high-risk groups. **(C, D)** Visualization of the enriched KEGG pathways. **(E)** GSEA of pathways in the hallmark gene set.

In the low-risk and high-risk groups, a total of 541 DEGs were identified, with 215 showing downregulation and 326 showing upregulation patterns as demonstrated by the volcano plot and heatmap in **Supplementary Figures 3A, B**. These DEGs were primarily associated with biological processes related to senescence-associated secretory phenotype (SASP), which has been proposed as a possible source of inflammatory factors in DKD caused by cellular senescence. Such SASP-associated biological processes include “POSITIVE REGULATION OF CYTOKINE PRODUCTION”, “TUMOR NECROSIS FACTOR PRODUCTION”, “CHEMOKINE PRODUCTION”, and “TRANSFORMING GROWTH FACTOR BETA PRODUCTION” (**Figure 6A**). Furthermore, pathway enrichment analysis revealed that DEGs were enriched in key pathways implicated in DKD pathogenesis, such as the “AGE-RAGE SIGNALING PATHWAY IN DIABETIC COMPLICATIONS”, “RENIN-ANGIOTENSIN SYSTEM”, “MAPK SIGNALING PATHWAY”, “PI3K-AKT SIGNALING PATHWAY”, and “TGF-beta SIGNALING PATHWAY”, among others (**Figures 6B–D**). We also conducted GSEA to identify underlying pathways or processes in hallmark gene sets obtained from the Molecular Signatures Database for patients with low or high scores. The results indicated that “EPITHELIAL MESENCHYMAL TRANSITION” and “TNF-α SIGNALING VIA NF-κb” were activated in the high-score group, while the activities of mitochondrial pathways, such as “FATTY ACID METABOLISM” and “OXIDATIVE PHOSPHORYLATION”, were suppressed (**Figure 6E**).

### 3.8 Evaluation of mitochondrial pathway

**Figure 7:**
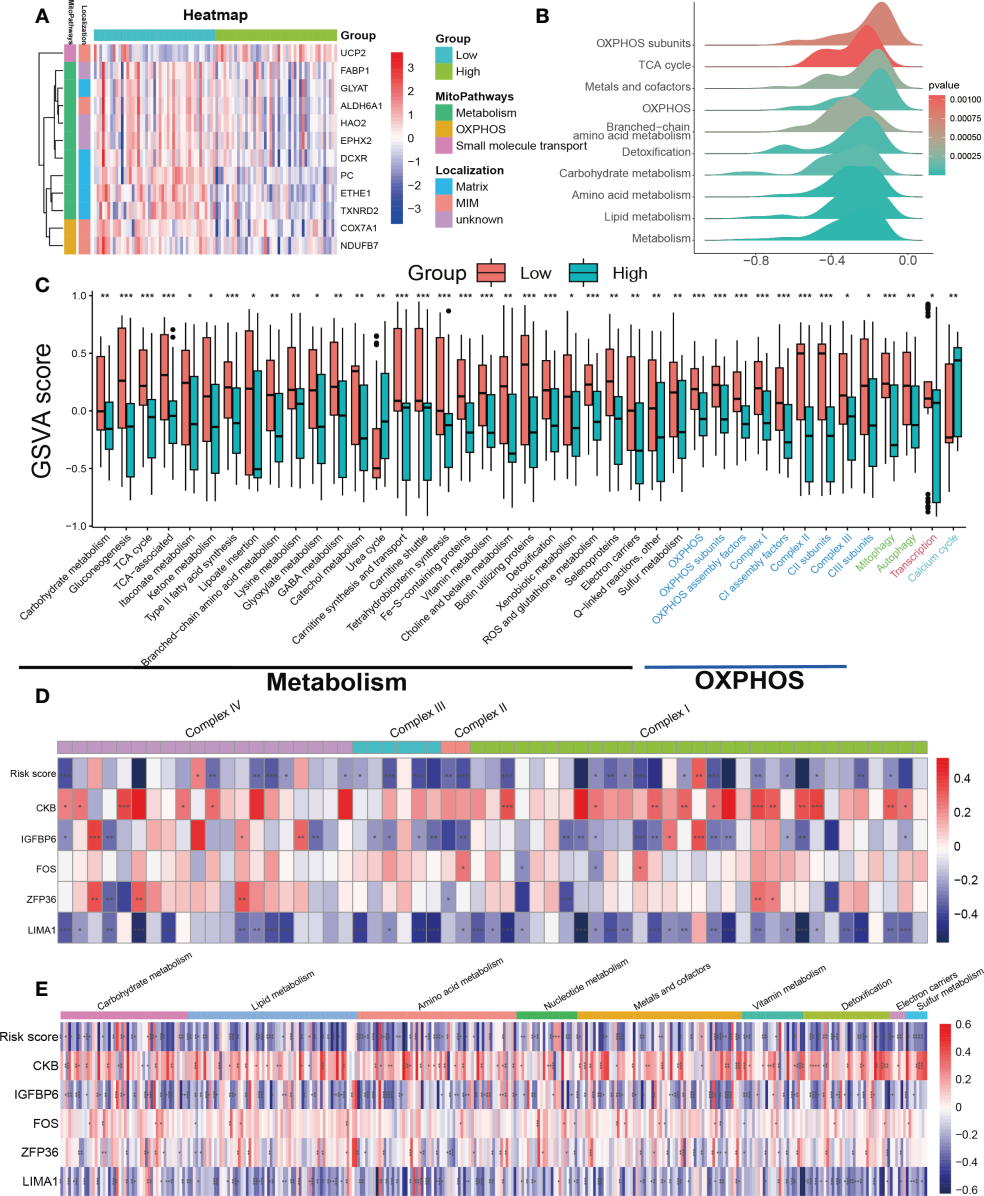
Assessment of mitochondrial pathways in the low-risk and high-risk groups. **(A)** Heatmap of differentially expressed mitochondrial genes in the low-risk and high-risk groups. **(B)** GSEA of mitochondria-related pathways in the low-risk and high-risk groups. **(C)** GSVA of mitochondrial pathways demonstrated by box plots in the low-risk and high-risk groups. **(D)** Correlations between the SRS risk score, the 5 hub genes and mitochondrial respiratory chain complex-related genes. **(E)** Correlations between the SRS risk score, the 5 hub genes and mitochondrial metabolism-related genes. * *p* < 0.05, ** *p* < 0.01, *** *p* < 0.001.

The previous analysis suggests that pathways related to mitochondrial function such as oxidative phosphorylation (OXPHOS) and fatty acid metabolism were significantly suppressed in the high SRS score group. Furthermore, mitochondrial dysfunction is a key hallmark of cellular senescence (26). Therefore, we further investigated the relationship between mitochondrial function and cellular senescence in our subsequent research. We obtained mitochondrial genes and pathways from the MitoCarta3.0 and Reactome database and assessed the differential expression of mitochondrial genes between the low- and high-score groups. Our analysis revealed that almost all differentially expressed mitochondrial genes were downregulated in the high SRS score group, primarily localized to the mitochondrial matrix and mitochondrial inner membrane (MIM) (**Figure 7A**). Based on the differential gene analysis between low- and high-score groups, we conducted GSEA enrichment analysis on mitochondrial pathways obtained from the MitoCarta3.0 database, indicating a significant suppression of pathways involved in OXPHOS and metabolism in the high SRS score group (**Figure 7B**). To further explore differences in mitochondrial pathways between the high- and low-score groups, we conducted GSVA analysis on mitochondrial pathways. We selected a log fold change threshold of 0.2 and a *p*-value below 0.05 to identify 41 pathways with significant differences, among which 39 pathways were downregulated in the high-score group (**Figure 7C**). Most of the downregulated pathways are related to OXPHOS and metabolism. In addition, the Mantel *t*-test confirmed an extremely strong correlation between the SRS score and the 5 hub genes with the OXPHOS and carbohydrate/lipid/amino acid metabolism gene sets (**Supplementary Figures 4A–D**). These findings suggest that mitochondrial function was widely suppressed in the high-score group.

To further investigate the mechanism behind the decreased activity of OXPHOS-related pathways in the high-scoring group, we analyzed the relationships between the expression of the SRS risk score, 5 hub genes, and genes related to the mitochondrial respiratory chain complex. As genes related to mitochondrial respiratory chain complex V were undetected in the expression profiles, we focused solely on genes related to complex I–IV. The correlation values revealed a clear negative correlation between the most respiratory chain complex I–IV genes and the SRS risk score, *LIMA1*, and *IGFBP6* expression levels, while *ZFP36*, *FOS*, and *CKB* showed a positive correlation (**Figure 7D**).

Continuing our investigation, we delved deeper into the correlation between the SRS score, 5 hub genes, and mitochondrial metabolism genes. Using the MitoCarta3.0 database, we obtained genes involved in mitochondrial metabolic pathways associated with various nutrient metabolisms. Our correlation analysis revealed a consistent regulatory effect of the SRS score and the 5 hub genes on genes regulating mitochondrial metabolism-related genes including macronutrients (carbohydrates/lipids/amino acids) and micronutrients (vitamins and minerals). Our findings indicated that the SRS risk score, *LIMA1*, and *IGFBP6* expression levels showed a negative correlation with most mitochondrial metabolism-related genes, while *ZFP36*, *FOS*, and *CKB* showed a positive correlation (**Figure 7E**).

Previous studies have suggested that both mitophagy and mitochondrial biogenesis play important roles in the senescence process (27, 28). Our GSVA indicated that the activity of the mitophagy pathway was decreased in the high-risk score group (**Figure 7C**). We downloaded gene sets related to PINK1/PRKN-mediated mitophagy, receptor-mediated mitophagy, and mitochondrial biogenesis from the Reactome database. Unfortunately, we did not find any clear patterns or correlations between the SRS risk score and the 5 hub genes with the genes related to the mitophagy gene sets (**Supplementary Figures 4E, F**). Regarding the gene sets related to mitochondrial biogenesis, we found that only *SIRT3* was negatively correlated with both the SRS risk score and the expression levels of *LIMA1*, *ZFP36*, *FOS*, and *IGFBP6*. Meanwhile, *CKB* showed a positive correlation with *SIRT3* expression. However, there was no significant correlation between the SRS risk score, the 5 hub genes, and other genes related to mitochondrial biogenesis as shown in **Supplementary Figure 4G**.

### 3.9 Evaluation of immune cell infiltration

**Figure 8:**
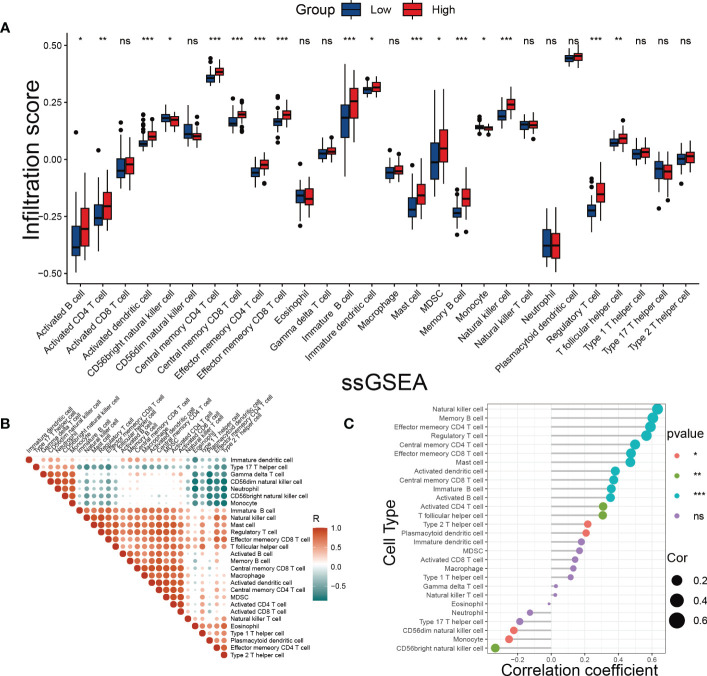
Evaluation of immune cell infiltration. **(A)** The score of immune cells was detected by ssGSEA grouped by the SRS risk score. **(B)** Correlation analysis between immune cells. **(C)** Correlation analysis between the SRS risk score and different immune cells. * *p* < 0.05, ** *p* < 0.01, *** *p* < 0.001. ns, no statistically significant difference.

One of the hallmarks of DKD is immune remodeling (29). To determine whether the SRS risk score accurately reflected the immune status of DKD, we evaluated immune cell infiltration in DKD using ssGSEA. We observed distinct immune infiltrate patterns among patient risk groups stratified by the SRS risk score. Compared to the low-risk group, most innate and adaptive immune cells showed higher levels of infiltration in the high-risk group (**Figure 8A**). Furthermore, there were notable interactions between immune cell populations across kidney tissues affected by DKD (**Figure 8B**). Correlation analysis revealed that infiltration levels of multiple types of immune cells, including T-cell subsets, different developmental or functional stages of B cells, and mast cells, were positively correlated with the SRS risk score, while CD56dim natural killer cells, monocytes, and CD56bright natural killer cells were negatively correlated with the risk score (**Figure 8C**). These findings suggest that immune system disorders in the process of DKD may be closely related to cellular senescence.

## 4 Discussion

Our study constructed an SRS risk score using 5 biomarkers related to cellular senescence based on the GEO datasets. We found that patients in the high-scoring group had lower GFR and higher expression of fibrotic genes compared to those in the low-scoring group. Additionally, we observed significant suppression of mitochondrial function in the high-scoring group. Notably, the SRS risk score was positively correlated with the degree of immune cell infiltration in DKD. These results provide new insight into the role of cellular senescence and associated genes in the pathogenesis of DKD.

Cellular senescence was first reported in the 1960s (30) and is defined as a persistent cell cycle arrest that limits their proliferative life span. Emerging evidence for accelerated senescence and SASP has been reported in DKD (3, 31). Recent studies have shown that drugs targeting senescent cells can alleviate proteinuria in obese insulin-resistant mice (32), suggesting that anti-cellular senescence strategies hold promise for future interventions aimed at delaying, preventing, or treating renal dysfunction. In our research, the 5 hub genes (*LIMA1*, *IGFBP6*, *ZFP36*, *FOS*, and *CKB*) related to cellular senescence were identified. Importantly, these hub genes have significant implications in multiple senescence-related fields. For example, *ZFP3*6 contributes to inflammation-associated lung damage by interacting with the p53/p21 pathway, which is a core pathway in senescence (33). In addition, *FOS* plays a crucial role in the senescence process of granulosa cells in the ovary (34), and is responsible for UV-related skin aging (35). *CKB* is involved in cigarette smoke-induced bronchial epithelial cell senescence (36). However, the roles of these hub genes in the regulation of cellular senescence in DKD are less well understood. It should be noted that further research is necessary to validate the effects of these identified genes in DKD.

Clear evidence indicates that AGEs formation generation plays a central role in DKD pathology mechanisms. The binding of AGEs with receptor for AGEs (RAGE) provokes oxidative stress and chronic inflammation in renal tissues, resulting in progressive renal diseases (37). These processes involve activations of signaling pathways nuclear factor-kappa B (NF-κB), PI3K/Akt, and MAPK/ERK (38). Our study found that the AGE/RAGE, PI3K/Akt, and ERK pathways were enriched in the DEGs grouped by the SRS risk score; these results suggest that cellular senescence is involved in the regulation of the core pathways in DKD.

Along with cellular senescence, mitochondrial dysfunction is an essential “hallmark” and drives and maintains cellular senescence (26, 39). However, there is still no consensus on which type of mitochondrial dysfunction is the primary driving factor of cellular senescence. Previous studies have emphasized that mitochondrial OXPHOS dysfunction is a common feature of senescent cells (40), and our study confirms this finding again in the DKD model. Furthermore, we have demonstrated that genes related to mitochondrial respiratory chain complexes are widely downregulated in patients with higher SRS score. Mitochondria integrate nutrients as fuels to generate energy for the cell (41), and the metabolic disorders of these nutrients are involved in the occurrence and development of DKD (42–44); however, few studies have suggested that nutrient metabolism disorder is closely related to cellular senescence in the process of DKD, and it is worth investigating whether targeting mitochondrial metabolism could potentially restore mitochondrial function during cellular senescence. Additionally, numerous studies have suggested that mitophagy plays a role in regulating cellular senescence and is involved in the development of DKD (31). Our GSVA indicated that the activity of the mitophagy pathway was decreased in the high SRS score group. Unfortunately, we did not find any clear patterns or correlations between our SRS risk score and the 5 hub genes with the genes related to the mitophagy gene sets. This may be due to the fact that the regulation of mitophagy often occurs at the post-transcriptional protein modification level (45, 46).

Although immune dysregulation has been recognized as a crucial driving factor in the pathogenesis of DKD, the potential regulation of the immune system remains largely unknown. Our study identified numerous immune-related biological processes and pathways that were significantly enriched in the differentially expressed DEGs grouped by the SRS risk score. Notably, most immune cells were increased in the high-risk score group, suggesting that cellular senescence may trigger an immunological response in DKD. Multiple studies have reported that immune cell infiltration contributes to the development of DKD. For instance, group 2 innate lymphoid cells have been implicated in the pathogenesis of renal fibrosis in DKD (47). The deposition of macrophages in the kidney is closely associated with decreased renal function in DKD patients (48), while mast cells are reported to participate in renal interstitial fibrosis during DKD progression (49). Memory CD8+ T cells were also found to be significantly elevated in kidney tissues in various types of chronic kidney disease (CKD), including DKD, leading to podocyte injury and glomerulosclerosis (50). Based on our findings, targeting senescent cells could potentially represent a novel therapeutic approach for improving immune dysfunction in DKD patients.

This study has several limitations. First, we built and evaluated our SRS from public databases. To confirm its clinical value, more prospective real-world evidence is needed. Second, the SRS construction based solely on a single signature is inescapable because many significant genes in DKD may have been ignored. Third, the association between SRS and mitochondrial pathway, immune response, needs to be studied experimentally.

In conclusion, an innovative SRS can distinguish normal control and DKD patients and can be utilized to predict kidney function. Moreover, the SRS was found to be linked to mitochondrial function and immune cell infiltration.

## Data availability statement

The datasets presented in this study can be found in online repositories. The names of the repository/repositories and accession number(s) can be found in the article/**Supplementary Material**.

## Ethics statement

The animal study was reviewed and approved by The Sixth Affiliated Hospital, Sun Yat-sen University.

## Author contributions

TZ designed and supervised the entire work. YL conceived the study and performed the literature search and bioinformatics analysis. LZ performed the experiments and wrote the manuscript. All authors contributed to the article and approved the submitted version.

## Supplementary material

Supplementary Figure 1 Flow chart of this study.

Supplementary Figure 2 Data preprocessing. **(A, C)** Box plots of datasets before **(A)** and after **(C)** data processing. **(B, D)** PCA of datasets before **(B)** and after **(D)** data processing.

Supplementary Figure 3 Identification of DEGs grouped by SRS. **(A, B)** Heatmap and the volcano plot of the DEGs from low-risk group and high-risk group.

Supplementary Figure 4 Assessment of mitochondrial pathways in the low-risk and high-risk groups. **(A)** Correlations between the SRS score, the 5 hub genes and OXPHOS, **(B–D)** carbohydrate/lipid/amino acid metabolism, **(E, F)** mitophagy, **(G)** mitochondrial biogenesis gene set.

## References

[1] Umanath K Lewis JB. Update on diabetic nephropathy: core curriculum 2018. Am J Kidney Dis (2018) 71(2018):884–95. doi: 10.1053/j.ajkd.2017.10.026 29398179

[2] Muñoz-Espín D Serrano M. Cellular senescence: from physiology to pathology. Nat Rev Mol Cell Biol (2014) 15:482–96. doi: 10.1038/nrm3823 24954210

[3] Jia C Ke-Hong C Fei X Huan-Zi D Jie Y Li-Ming W. Decoy receptor 2 mediation of the senescent phenotype of tubular cells by interacting with peroxiredoxin 1 presents a novel mechanism of renal fibrosis in diabetic nephropathy. Kidney Int (2020) 98:645–62. doi: 10.1016/j.kint.2020.03.026 32739204

[4] Liu J Yang JR Chen XM Cai GY Lin LR He YN. Impact of ER stress-regulated ATF4/p16 signaling on the premature senescence of renal tubular epithelial cells in diabetic nephropathy. Am J Physiol Cell Physiol (2015) 308:C621–30. doi: 10.1152/ajpcell.00096.2014 25567807

[5] Prattichizzo F De Nigris V Mancuso E Spiga R Giuliani A Matacchione G. Short-term sustained hyperglycaemia fosters an archetypal senescence-associated secretory phenotype in endothelial cells and macrophages. Redox Biol (2018) 15:170–81. doi: 10.1016/j.redox.2017.12.001 PMC5735298 29253812

[6] Liberzon A Birger C Thorvaldsdóttir H Ghandi M Mesirov JP Tamayo P. The molecular signatures database (MSigDB) hallmark gene set collection. Cell Syst (2015) 1:417–25. doi: 10.1016/j.cels.2015.12.004 PMC4707969 26771021

[7] Avelar RA Ortega JG Tacutu R Tyler EJ Bennett D Binetti P. A multidimensional systems biology analysis of cellular senescence in aging and disease. Genome Biol (2020) 21:91. doi: 10.1186/s13059-020-01990-9 32264951 PMC7333371

[8] Eddy S Mariani LH Kretzler M. Integrated multi-omics approaches to improve classification of chronic kidney disease. Nat Rev Nephrol (2020) 16:657–68. doi: 10.1038/s41581-020-0286-5 32424281

[9] Rath S Sharma R Gupta R Ast T Chan C Durham TJ. MitoCarta3.0: an updated mitochondrial proteome now with sub-organelle localization and pathway annotations. Nucleic Acids Res (2021) 49:D1541–d1547. doi: 10.1093/nar/gkaa1011 33174596 PMC7778944

[10] Gillespie M Jassal B Stephan R Milacic M Rothfels K Senff-Ribeiro A. The reactome pathway knowledgebase 2022. Nucleic Acids Res (2022) 50:D687–d692. doi: 10.1093/nar/gkab1028 34788843 PMC8689983

[11] Parker HS Leek JT Favorov AV Considine M Xia X Chavan S. Preserving biological heterogeneity with a permuted surrogate variable analysis for genomics batch correction. Bioinf (Oxford England) (2014) 30:2757–63. doi: 10.1093/bioinformatics/btu375 PMC4173013 24907368

[12] Kanehisa M Sato Y Kawashima M Furumichi M Tanabe M. KEGG as a reference resource for gene and protein annotation. Nucleic Acids Res (2016) 44:D457–62. doi: 10.1093/nar/gkv1070 PMC4702792 26476454

[13] The gene ontology resource: 20 years and still GOing strong. Nucleic Acids Res (2019) 47:D330–d338. doi: 10.1093/nar/gky1055 30395331 PMC6323945

[14] Yu G Wang LG Han Y He QY. clusterProfiler: an r package for comparing biological themes among gene clusters. Omics J Integr Biol (2012) 16:284–7. doi: 10.1089/omi.2011.0118 PMC3339379 22455463

[15] Subramanian A Tamayo P Mootha VK Mukherjee S Ebert BL Gillette MA. Gene set enrichment analysis: a knowledge-based approach for interpreting genome-wide expression profiles. Proc Natl Acad Sci USA (2005) 102:15545–50. doi: 10.1073/pnas.0506580102 PMC1239896 16199517

[16] Hänzelmann S Castelo R Guinney J. GSVA: gene set variation analysis for microarray and RNA-seq data. BMC Bioinf (2013) 14:7. doi: 10.1186/1471-2105-14-7 PMC3618321 23323831

[17] Langfelder P Horvath S. WGCNA: an r package for weighted correlation network analysis. BMC Bioinf (2008) 9:559. doi: 10.1186/1471-2105-9-559 PMC2631488 19114008

[18] Ritchie ME Phipson B Wu D Hu Y Law CW Shi W. Limma powers differential expression analyses for RNA-sequencing and microarray studies. Nucleic Acids Res (2015) 43:e47. doi: 10.1093/nar/gkv007 25605792 PMC4402510

[19] Ito K Murphy D. Application of ggplot2 to pharmacometric graphics. CPT: Pharmacometrics Syst Pharmacol (2013) 2:e79. doi: 10.1038/psp.2013.56 24132163 PMC3817376

[20] Lin X Yang F Zhou L Yin P Kong H Xing W. A support vector machine-recursive feature elimination feature selection method based on artificial contrast variables and mutual information. J chromatography. B Analytical Technol Biomed Life Sci (2012) 910:149–55. doi: 10.1016/j.jchromb.2012.05.020 22682888

[21] Guo L Wang Z Du Y Mao J Zhang J Yu Z. Random-forest algorithm based biomarkers in predicting prognosis in the patients with hepatocellular carcinoma. Cancer Cell Int (2020) 20:251. doi: 10.1186/s12935-020-01274-z 32565735 PMC7302385

[22] Huang ML Hung YH Lee WM Li RK Jiang BR. SVM-RFE based feature selection and taguchi parameters optimization for multiclass SVM classifier. TheScientificWorldJournal (2014) 2014:795624. doi: 10.1155/2014/795624 PMC4175386 25295306

[23] Sing T Sander O Beerenwinkel N Lengauer T. ROCR: visualizing classifier performance in r. Bioinf (Oxford England) (2005) 21:3940–1. doi: 10.1093/bioinformatics/bti623 16096348

[24] Zhao E Xie H Zhang Y. Predicting diagnostic gene biomarkers associated with immune infiltration in patients with acute myocardial infarction. Front Cardiovasc Med (2020) 7:586871. doi: 10.3389/fcvm.2020.586871 33195475 PMC7644926

[25] Han YC Tang SQ Liu YT Li AM Zhan M Yang M. AMPK agonist alleviate renal tubulointerstitial fibrosis. via activating mitophagy High fat streptozotocin induced Diabetic mice. Cell Death Dis (2021) 12:925. doi: 10.1038/s41419-021-04184-8 34628484 PMC8502176

[26] López-Otín C Blasco MA Partridge L Serrano M Kroemer G. The hallmarks of aging. Cell (2013) 153:1194–217. doi: 10.1016/j.cell.2013.05.039 PMC3836174 23746838

[27] Popov LD. Mitochondrial biogenesis: an update. J Cell Mol Med (2020) 24:4892–9. doi: 10.1111/jcmm.15194 PMC7205802 32279443

[28] Korolchuk VI Miwa S Carroll B von Zglinicki T. Mitochondria in cell senescence: is mitophagy the weakest link? EBioMedicine (2017) 21:7–13. doi: 10.1016/j.ebiom.2017.03.020 28330601 PMC5514379

[29] Fu J Sun Z Wang X Zhang T Yuan W Salem F. The single-cell landscape of kidney immune cells reveals transcriptional heterogeneity in early diabetic kidney disease. Kidney Int (2022) 102:1291–304. doi: 10.1016/j.kint.2022.08.026 PMC9691617 36108806

[30] Hayflick L Moorhead PS. The serial cultivation of human diploid cell strains. Exp Cell Res (1961) 25:585–621. doi: 10.1016/0014-4827(61)90192-6 13905658

[31] Chen K Dai H Yuan J Chen J Lin L Zhang W. Optineurin-mediated mitophagy protects renal tubular epithelial cells against accelerated senescence in diabetic nephropathy. Cell Death Dis (2018) 9:105. doi: 10.1038/s41419-017-0127-z 29367621 PMC5833650

[32] Palmer AK Xu M Zhu Y Pirtskhalava T Weivoda MM Hachfeld CM. Targeting senescent cells alleviates obesity-induced metabolic dysfunction. Aging Cell (2019) 18:e12950. doi: 10.1111/acel.12950 30907060 PMC6516193

[33] Cao Y Huang W Wu F Shang J Ping F Wang W. ZFP36 protects lungs from intestinal I/R-induced injury and fibrosis through the CREBBP/p53/p21/Bax pathway. Cell Death Dis (2021) 12:685. doi: 10.1038/s41419-021-03950-y 34238924 PMC8266850

[34] Jiang ZX Wang YN Li ZY Dai ZH He Y Chu K. The m6A mRNA demethylase FTO in granulosa cells retards FOS-dependent ovarian aging. Cell Death Dis (2021) 12:744. doi: 10.1038/s41419-021-04016-9 34315853 PMC8316443

[35] Angel P Szabowski A Schorpp-Kistner M. Function and regulation of AP-1 subunits in skin physiology and pathology. Oncogene (2001) 20:2413–23. doi: 10.1038/sj.onc.1204380 11402337

[36] Hara H Araya J Takasaka N Fujii S Kojima J Yumino Y. Involvement of creatine kinase b in cigarette smoke-induced bronchial epithelial cell senescence. Am J Respir Cell Mol Biol (2012) 46:306–12. doi: 10.1165/rcmb.2011-0214OC 21980054

[37] Molitch ME Adler AI Flyvbjerg A Nelson RG So WY Wanner C. Diabetic kidney disease: a clinical update from kidney disease: improving global outcomes. Kidney Int (2015) 87:20–30. doi: 10.1038/ki.2014.128 24786708 PMC4214898

[38] Wu XQ Zhang DD Wang YN Tan YQ Yu XY Zhao YY. AGE/RAGE in diabetic kidney disease and ageing kidney. Free Radical Biol Med (2021) 171:260–71. doi: 10.1016/j.freeradbiomed.2021.05.025 34019934

[39] Correia-Melo C Marques FD Anderson R Hewitt G Hewitt R Cole J. Mitochondria are required for pro-ageing features of the senescent phenotype. EMBO J (2016) 35:724–42. doi: 10.15252/embj.201592862 PMC4818766 26848154

[40] Miwa S Kashyap S Chini E von Zglinicki T. Mitochondrial dysfunction in cell senescence and aging. J Clin Invest (2022) 132. doi: 10.1172/jci158447 PMC9246372 35775483

[41] Spinelli JB Haigis MC. The multifaceted contributions of mitochondria to cellular metabolism. Nat Cell Biol (2018) 20:745–54. doi: 10.1038/s41556-018-0124-1 PMC6541229 29950572

[42] Fu Y Sun Y Wang M Hou Y Huang W Zhou D. Elevation of JAML promotes diabetic kidney disease by modulating podocyte lipid metabolism. Cell Metab (2020) 32:1052–1062.e8. doi: 10.1016/j.cmet.2020.10.019 33186558

[43] Ansermet C Centeno G Bignon Y Ortiz D Pradervand S Garcia A. Dysfunction of the circadian clock in the kidney tubule leads to enhanced kidney gluconeogenesis and exacerbated hyperglycemia in diabetes. Kidney Int (2022) 101:563–73. doi: 10.1016/j.kint.2021.11.016 34838539

[44] Liu L Xu J Zhang Z Ren D Wu Y Wang D. Metabolic homeostasis of amino acids and diabetic kidney disease. Nutrients (2022) 15. doi: 10.3390/nu15010184 PMC9823842 36615841

[45] Heo JM Ordureau A Swarup S Paulo JA Shen K Sabatini DM. RAB7A phosphorylation by TBK1 promotes mitophagy via the PINK-PARKIN pathway. Sci Adv (2018) 4:eaav0443. doi: 10.1126/sciadv.aav0443 30627666 PMC6314648

[46] Yao J Wang J Xu Y Guo Q Sun Y Liu J. CDK9 inhibition blocks the initiation of PINK1-PRKN-mediated mitophagy by regulating the SIRT1-FOXO3-BNIP3 axis and enhances the therapeutic effects involving mitochondrial dysfunction in hepatocellular carcinoma. Autophagy (2022) 18:1879–97. doi: 10.1080/15548627.2021.2007027 PMC9450969 34890308

[47] Liu C Qin L Ding J Zhou L Gao C Zhang T. Group 2 innate lymphoid cells participate in renal fibrosis in diabetic kidney disease partly. via TGF-β1 Signal Pathway. J Diabetes Res (2019) 2019:8512028. doi: 10.1155/2019/8512028 31355294 PMC6636594

[48] Nguyen D Ping F Mu W Hill P Atkins RC Chadban SJ. Macrophage accumulation in human progressive diabetic nephropathy. Nephrol (Carlton Vic.) (2006) 11:226–31. doi: 10.1111/j.1440-1797.2006.00576.x 16756636

[49] Okoń K Stachura J. Increased mast cell density in renal interstitium is correlated with relative interstitial volume, serum creatinine and urea especially in diabetic nephropathy but also in primary glomerulonephritis. Polish J Pathol Off J Polish Soc Pathologists (2007) 58:193–7. 18074865

[50] Li L Tang W Zhang Y Jia M Wang L Li Q. Targeting tissue-resident memory CD8(+) T cells in the kidney is a potential therapeutic strategy to ameliorate podocyte injury and glomerulosclerosis. Mol Ther (2022) 30:2746–59. doi: 10.1016/j.ymthe.2022.04.024 PMC9372318 35514086

