# Supplementary figures and images for: Identification and analysis of cellular senescence-associated signatures in diabetic kidney disease by integrated bioinformatics analysis and machine learning
Source: Front Endocrinol (Lausanne). 2023 Jun 16;14:1193228. doi: 10.3389/fendo.2023.1193228 (PMC10313062; doi:10.3389/fendo.2023.1193228)

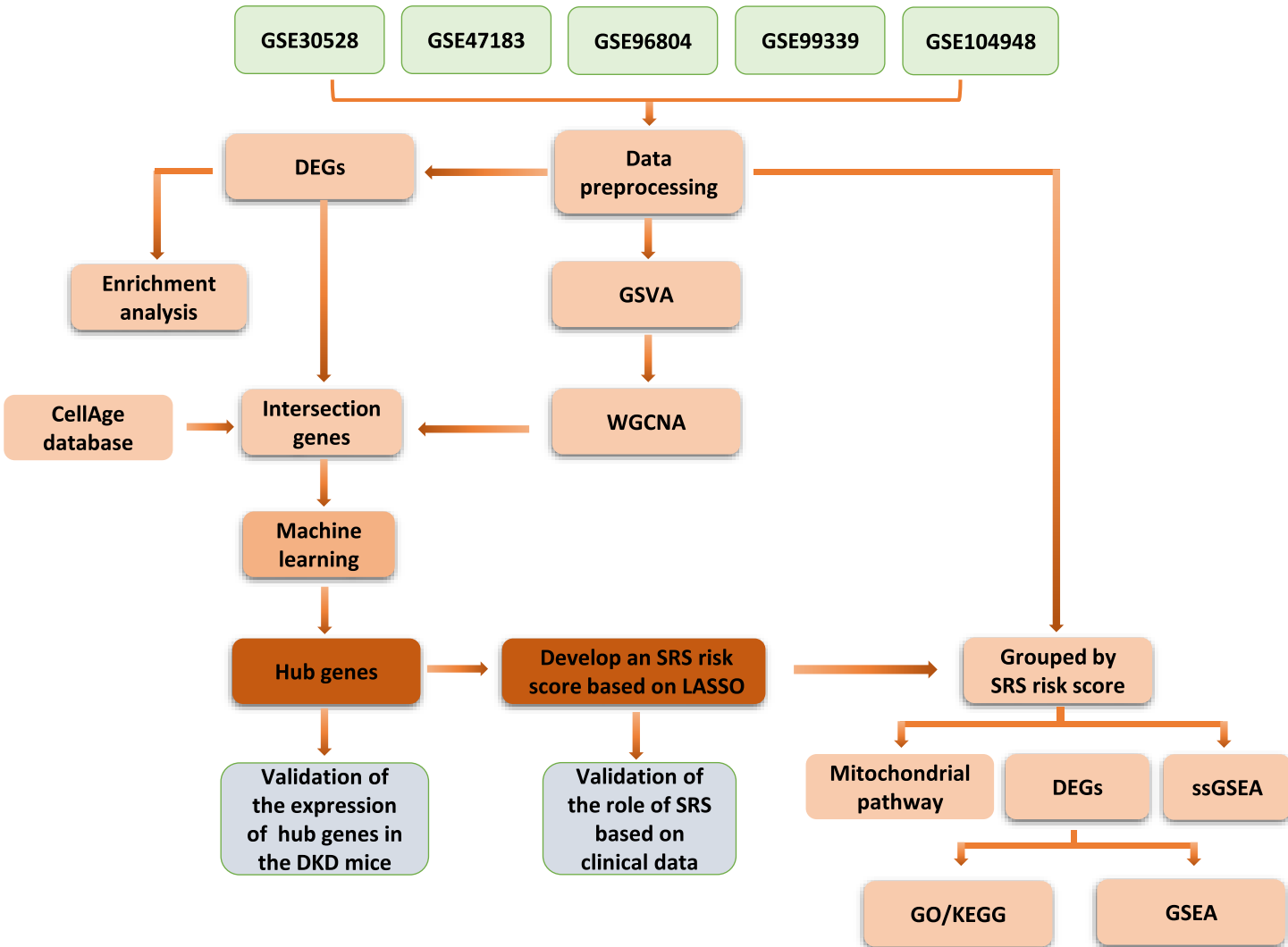

Supplement: Supplementary Figure 1 — Flow chart of this study. [file Image_1.pdf]

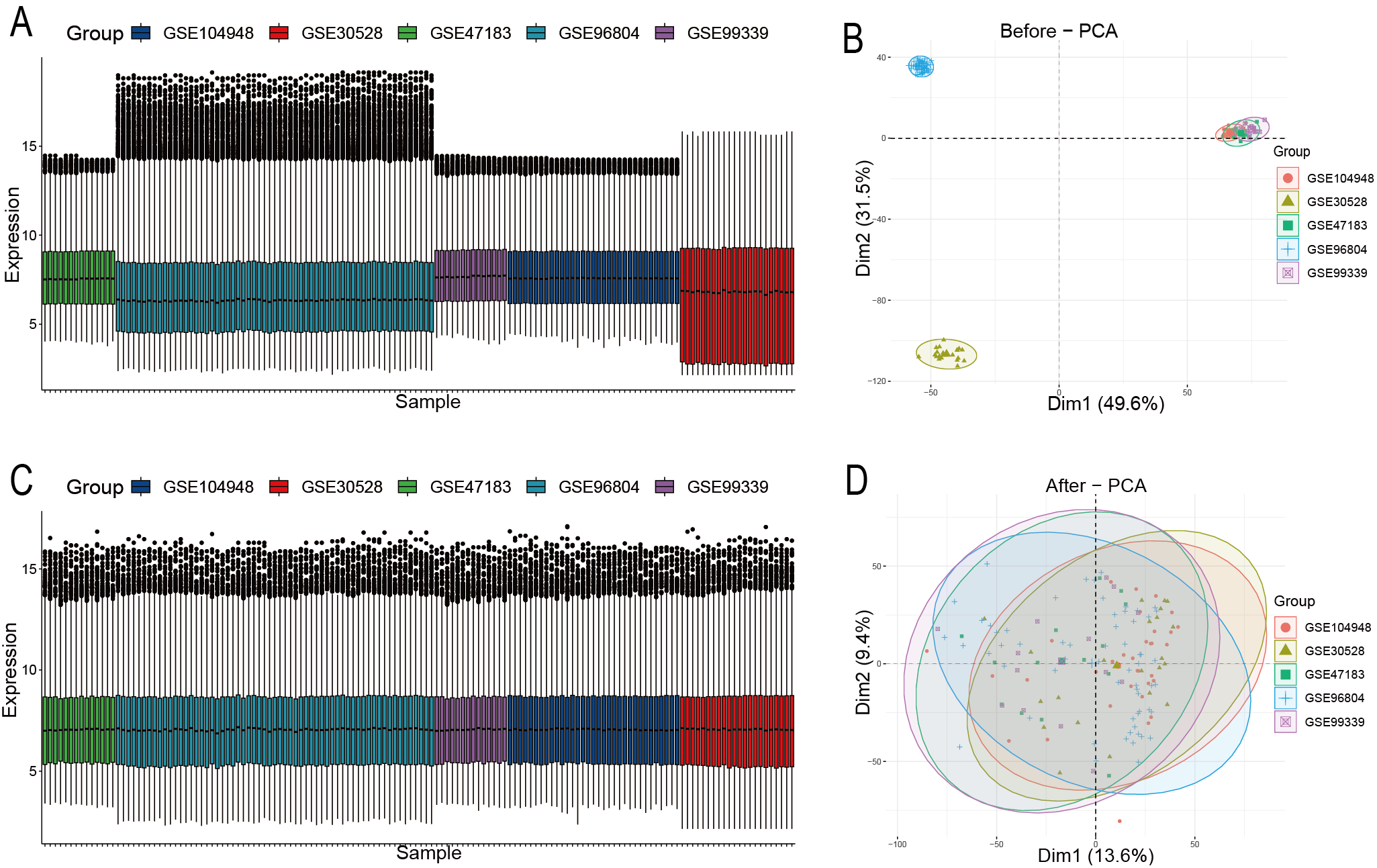

Supplement: Supplementary Figure 2 — Data preprocessing. (A, C) Box plots of datasets before (A) and after (C) data processing. (B, D) PCA of datasets before (B) and after (D) data processing. [file Image_2.tif]

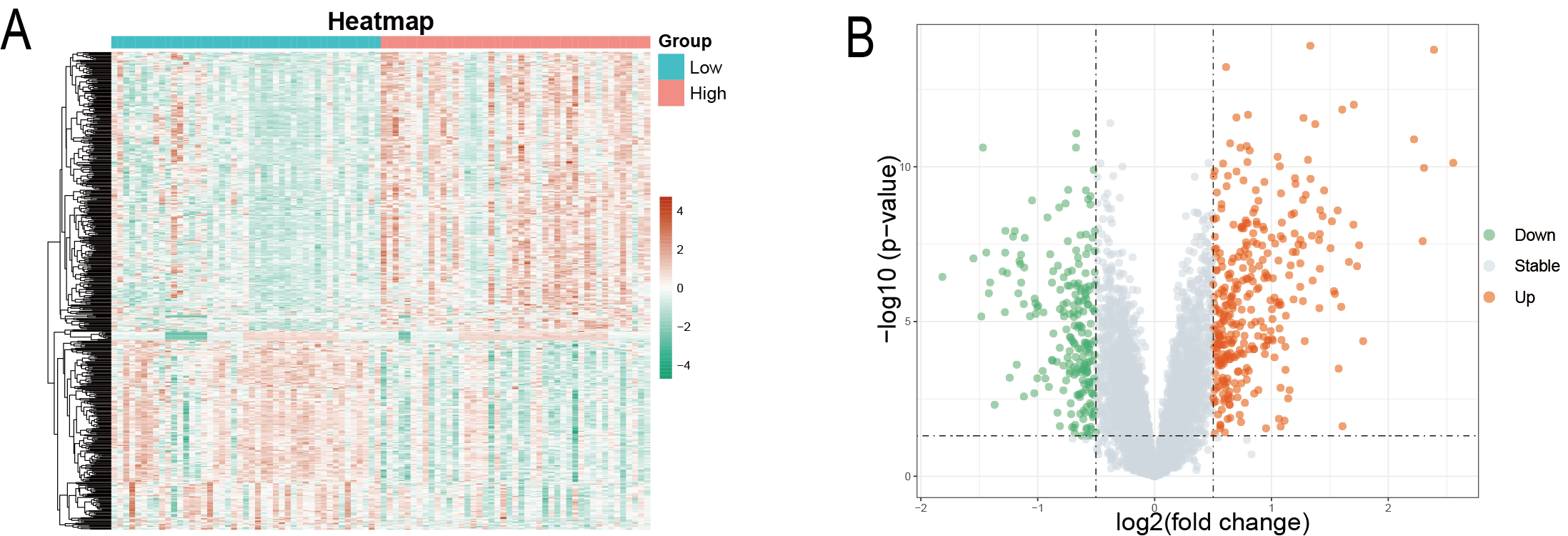

Supplement: Supplementary Figure 3 — Identification of DEGs grouped by SRS. (A, B) Heatmap and the volcano plot of the DEGs from low-risk group and high-risk group. [file Image_3.tif]

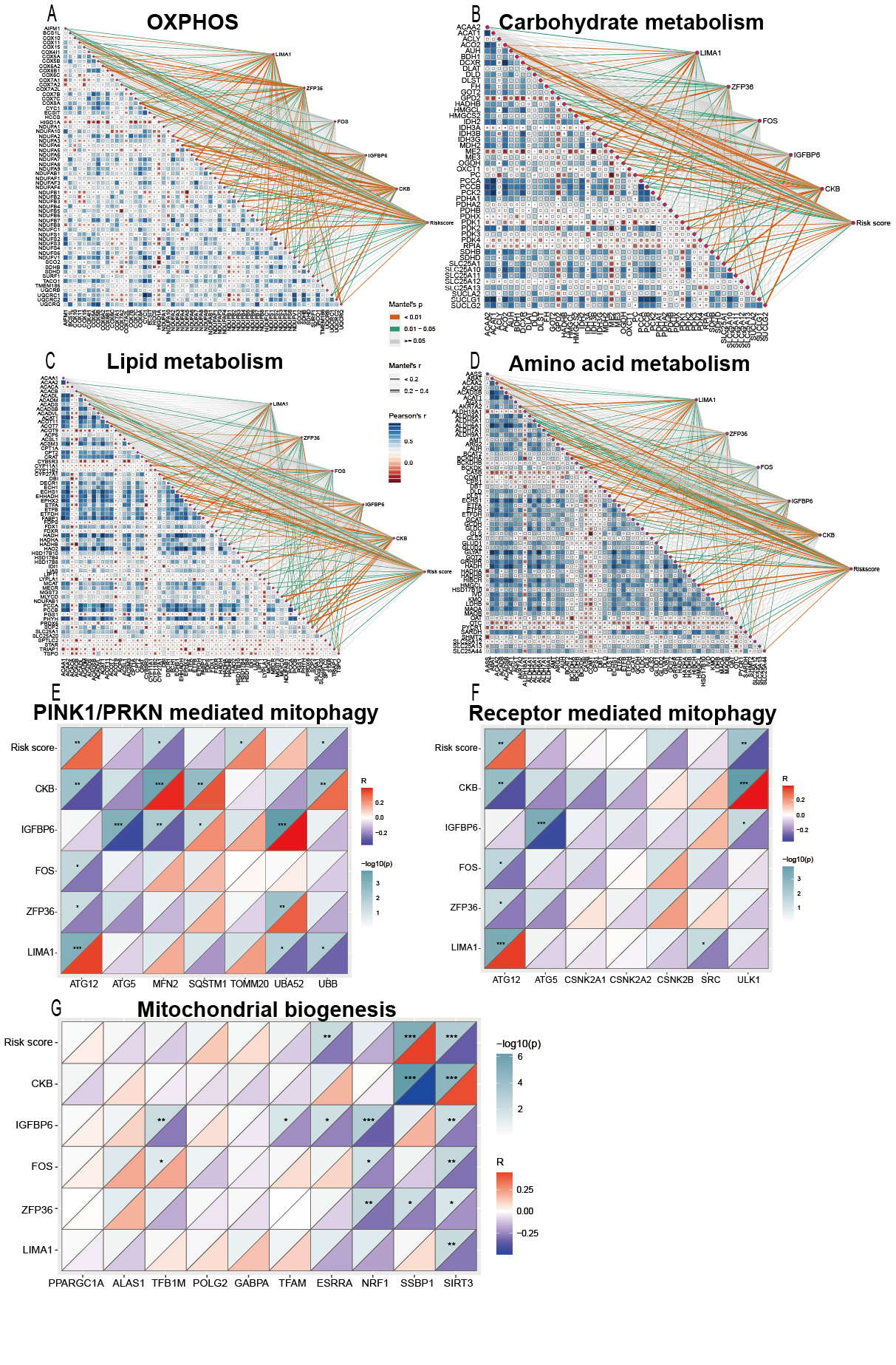

Supplement: Supplementary Figure 4 — Assessment of mitochondrial pathways in the low-risk and high-risk groups. (A) Correlations between the SRS score, the 5 hub genes and OXPHOS, (B–D) carbohydrate/lipid/amino acid metabolism, (E, F) mitophagy, (G) mitochondrial biogenesis gene set. [file Image_4.tif]
