# Supplementary material for: Identification and analysis of cellular senescence-associated signatures in diabetic kidney disease by integrated bioinformatics analysis and machine learning
Source: Front Endocrinol (Lausanne). 2023 Jun 16;14:1193228. doi: 10.3389/fendo.2023.1193228 (PMC10313062; doi:10.3389/fendo.2023.1193228)
Supplement: Supplementary file 5 [file Table_1.docx]

**Supplementary Table 1.** The essential information of included microarray datasets in this study.

| **GEO** | **Samples** | | |
| --- | --- | --- | --- |
|  | **Total** | **DKD** | **Control** |
| GSE30528 | 22 | 9 | 13 |
| GSE47183 | 14 | 14 | 0 |
| GSE96804 | 61 | 41 | 20 |
| GSE99339 | 14 | 14 | 0 |
| GSE104948 | 33 | 12 | 21 |
