# Supplementary material for: Identification and analysis of cellular senescence-associated signatures in diabetic kidney disease by integrated bioinformatics analysis and machine learning
Source: Front Endocrinol (Lausanne). 2023 Jun 16;14:1193228. doi: 10.3389/fendo.2023.1193228 (PMC10313062; doi:10.3389/fendo.2023.1193228)
Supplement: Supplementary file 6 [file Table_2.docx]

**Supplementary Table 2.** Sequences of primers used in RT-PCR analysis.

| **Gene** | **Forward primer** | **Reverse primer** |
| --- | --- | --- |
| M-*LIMA1* | GTTTCACATCAGCTGTTTCCGA | GCCCTCGTCATAGTTGCCTT |
| M-*ZFP36* | CCTCAGGCCGCAGAAGCTC | TCTCTTCGAGTCACAGGGGT |
| M-*FOS* | GCCAGTCAAGAGCATCAGCAAC | CATCCCCAAGGAATTGCTGT |
| M-*IGFBP6* | CCTGCAGACGGCTGTACAGA | CCTCTTCGTTCTCTCGGGGT |
| M-*CKB* | TTTGATGTCTCCAACGCTGACC | TCATCGATTGCCTGACCCTG |
| M-*β-ACTIN* | TATGCTCTCCCTCACGCCAT | GCACGATTTCCCTCTCAGCT |

**References**
